# Iterative Min Cut Clustering Based on Graph Cuts

**DOI:** 10.3390/s21020474

**Published:** 2021-01-11

**Authors:** Bowen Liu, Zhaoying Liu, Yujian Li, Ting Zhang, Zhilin Zhang

**Affiliations:** 1 Faculty of Information Technology, Beijing University of Technology, Beijing 100124, China; liubw2017@emails.bjut.edu.cn (B.L.); zhangting@bjut.edu.cn (T.Z.); zhangzl@emails.bjut.edu.cn (Z.Z.); 2 School of Artificial Intelligence, Guilin University of Electronic Technology, Guilin 541004, China; liyujian@guet.edu.cn

**Keywords:** clustering, graph cuts, variational method, partial differential equation, nonlinearly separable datasets

## Abstract

Clustering nonlinearly separable datasets is always an important problem in unsupervised machine learning. Graph cut models provide good clustering results for nonlinearly separable datasets, but solving graph cut models is an NP hard problem. A novel graph-based clustering algorithm is proposed for nonlinearly separable datasets. The proposed method solves the min cut model by iteratively computing only one simple formula. Experimental results on synthetic and benchmark datasets indicate the potential of the proposed method, which is able to cluster nonlinearly separable datasets with less running time.

## 1. Introduction

Clustering algorithms classify data points into *C* clusters (or categories) on the basis of their similarity. Its applications range from image processing [1,2] to biology [3], sociology [4], and business [5]. Clustering algorithms mainly include partition-based clustering [6,7,8,9], density-based clustering [10,11], and graph-based clustering [12,13,14], etc. In partition-based clustering algorithms, the mean (or median) of a cluster is viewed as the clustering center, and a data point is assigned to the nearest center. In density-based clustering algorithms, clusters are groups of data points characterized by the same local density, and a clustering center is the data point of which local density is higher. Graph-based clustering algorithms define a graph with vertices equal to the elements of a dataset, and edges are weighted by the similarity between pairs of data points in the dataset. Then the algorithms find an optimal partition of the graph such that the edges between different subgraph have a very low weight and the edges within a subgraph have high weight. There are several popular constructions to transform a dataset into a similarity graph, such as k-nearest neighbor (KNN) graph and mutual k-nearest neighbor (MKNN) graph [12]. The commonly used graph cut criterions include min cut, ratio cut, normalized cut (Ncut) and Cheeger cut.

Clustering nonlinearly separable datasets is a challenging problem in clustering analysis. Many methods have been proposed to solve this problem. Kernel method maps a nonlinearly separable dataset into a higher-dimensional Hilbert space, and in the Hilbert space the dataset may be linearly separable. DBK clustering [15] proposes a density equalization principle, and then based on this principle, they propose an adaptive kernel clustering algorithm. Multiple kernels clustering algorithms [16,17,18,19] use multiple kernel functions to enhance the performance of kernel clustering algorithms. Kernel K-means (or Kernel fuzzy K-means) algorithms with appropriate kernel functions are able to cluster nonlinearly separable datasets, but it is difficult to select appropriate kernel functions.

Spectral clustering, which is a famous graph-based clustering algorithm, firstly constructs a graph Laplacian matrix, and then computes eigenvalues and eigenvectors of the graph Laplacian matrix. It regards eigenvectors corresponding to the *k* smallest eigenvalues as low-dimensional embeddings of the dataset, and finally uses some basic clustering algorithms (for example, K-means) to obtain a clustering result. Hyperplanes clustering method [20] sets up a hyperplane framework to solve the Ncut problem. Sparse subspace clustering [21] builds a similarity graph by sparse representation techniques, and then uses spectral clustering to compute clustering results. Subspace Clustering by Block Diagonal Representation (BDR) [22] proposes a theory of block diagonal property, and is then based on the theory to build the similarity graph. Spectral clustering provides good clustering results for nonlinearly separable datasets, but it is complex to compute eigenvalues and eigenvectors.

In this article, a simple but effective clustering algorithm (called iterative min cut clustering) for nonlinearly separable datasets is proposed. The proposed method is based on graph cuts theory, and it does not require computing the Laplacian matrix, eigenvalues, and eigenvectors. The proposed iterative min cut clustering uses only one formula to map a nonlinearly separable dataset to a linearly separable one-dimensional representation. We demonstrate the performance of the proposed method on synthetic and real datasets.

The remainder of this article is organized as follows. Section 2 introduces the proposed iterative min cut (IMC) algorithm. Section 3 presents the experimental results on nonlinearly separable datasets. Finally, concluding remarks are given in Section 4.

### 1.1. Related Works

Graph cuts clustering partitions a dataset $X=\left\{x_{1},\cdots ,x_{N}\right\}\;\subset R^{H}$ into *C* clusters by constructing a graph and finding a partition of the graph such that vertexes (a data point is seen as a vertex of the graph) in same subgraph are similar to each vertex and vertexes in different subgraph are dissimilar from each vertex. The construction methods of transforming a data into a graph mainly include

(1) ε-neighborhood graph. It connects all vertexes (data points) whose pairwise distances are smaller than ε, and then obtains an undirected graph.
(2) K-nearest neighbor graphs. It connects a vertex $v_{i}$ and a vertex $v_{j}$ if $v_{i}$ is among the K-nearest neighbors of $v_{j}$ or if $v_{j}$ is among the K-nearest neighbors of $v_{i}$ (or if both $v_{i}$ is among the K-nearest neighbors of $v_{j}$ and $v_{j}$ is among the K-nearest neighbors of $v_{i}$).
(3) The fully connected graph. It connects all points, and then obtains a fully connected graph.

Graph cuts problem is an NP hard problem, and spectral clustering is the most popular method to solve this problem. The spectral clustering algorithm is detailed in Algorithm 1.
**Algorithm 1:** Spectral clustering.**Input:**X**Do:**      (1) Compute W where $w_{ij}$ is the similarity between $x_{i}$ and $x_{j}$,      and $w_{ij}$ is usually computed by $w_{ij}=\exp\left(-\frac{{∥x_{i}-x_{j}∥}^{2}}{2\sigma^{2}}\right)$      (2) Compute the Laplacian matrix L=D−W where D is the degree matrix,      and $d_{ij}$ is computed by $d_{i}=\sum_{j}w_{ij}$      (3) Compute the first *k* eigenvectors of L, and these eigenvectors are seen as low      dimensiona embedding of the original dataset      (4) Using K-means to cluster the low dimensional embedding**Output:** Clustering results of K-means

Spectral clustering provides good clustering results for nonlinearly separable datasets, but it requires to compute eigenvectors and eigenvalues of the Laplace matrix L. The cost of computing eigenvectors and eigenvalues is high without built-in tool.

## 2. Iterative Min Cut Clustering

In this section, we propose an iterative min cut clustering (IMC). The proposed IMC clustering algorithm partitions a dataset $X=\left\{x_{1},\cdots ,x_{N}\right\}\;\subset R^{H}$ into *C* clusters by minimizing the following objective function

(1) $$\sum_{i,j}w_{ij},\;x_{i}\;\mathrm{and}\;x_{j}\;\mathrm{belong}\;\mathrm{to}\;\mathrm{different}\;\mathrm{clusters}$$

where $w_{ij}$ is the similarity (i.e., the edge weight) between $x_{i}$ and $x_{j}$. For computational convenience, we normalize the data point $x_{i}$ as follows. For any $i\in \left\{1,\cdots ,N\right\}$,

(2) $$x_{i}=\frac{x_{i}}{\max\left\{x_{i}\left[1\right],\cdots ,x_{i}\left[H\right]\right\}}$$

The similarity $w_{ij}$ is computed by

(3) $$w_{ij}=\left\{\begin{array}{cc} \exp(-\frac{{∥x_{i}-x_{j}∥}^{2}}{2\sigma^{2}}), & x_{i}\;\mathrm{and}\;x_{j}\;\mathrm{are}\;\mathrm{neighbors}\\ 0, & \mathrm{otherwise} \end{array}\right..$$

We can use ε-neighborhood graph or K-nearest neighbor graphs (shown in Section 1.1) to select neighbors.

To solve (1), we define a feature *f* (*f* is a scalar) for each data point. If two data points belong to the same cluster, then their *f* values are the same. If two data points belong to the different cluster, then their *f* values are different. Let $f_{i}$ represent the feature of $x_{i}$. $f_{i}=f_{j}$ if $x_{i}$ and $x_{j}$ belong to the same cluster, and $f_{i}\neq f_{j}$ otherwise. $f=\left[f_{i}\right]={\left[f_{1},\cdots ,f_{N}\right]}^{T}$ can be viewed as a one-dimensional embedding of the dataset ***X***. (1) is equivalent to the following function

(4) $$J=\sum_{i=1}^{N}\sum_{j=1}^{N}w_{ij}{\left(f_{i}-f_{j}\right)}^{2}.$$

According to [12], we get the relationship between (4) and the Laplacian matrix L, i.e.,

(5) $$f^{T}Lf=\frac{1}{2}\sum_{i,j}w_{ij}{\left(f_{i}-f_{j}\right)}^{2}.$$

The problem $\min\sum_{i,j}w_{ij}{\left(f_{i}-f_{j}\right)}^{2}$ is equivalent to $\min f^{T}Lf$. By the Rayleigh–Ritz theorem [23], eigenvectors and eigenvalues of the matrix $f^{T}Lf$ are approximately equal to those of L, so spectral clustering computes eigenvectors of L instead of computing eigenvectors of $f^{T}Lf$. In this article, we use a novel solution to solve problem (4).

According to (4), we have for every $i\in \left\{1,\cdots ,N\right\}$ that

(6) $$\begin{array}{c} \frac{\partial J}{\partial f_{i}}=2\sum_{j}\left(f_{i}-f_{j}\right)w_{ij}-2\sum_{j}\left(f_{j}-f_{i}\right)w_{ji}\\ =4\sum_{j}\left(f_{i}-f_{j}\right)w_{ij} \end{array}.$$

Equating all the previous partial derivatives to zero (i.e., $\frac{\partial J}{\partial f_{i}}=0,i\in \left\{1,\cdots ,N\right\}$), we obtain the following values of $f_{i}$, for every $i\in \left\{1,\cdots ,N\right\}$

(7) $$f_{i}=\frac{\sum_{j}w_{ij}f_{j}}{\sum_{j}w_{ij}}.$$

According to variational method [24], (7) contains two *f*, and we can view a *f* as $f^{\left(k\right)}$, and view the other *f* as $f^{\left(k+1\right)}$. The proposed ideal is from variational method. The variational method is well supported by the theory, so the proposed method is indirectly supported by the theory of variational method. The proposed method uses only one formula to solve the problem (4) (Spectral clustering requires computing eigenvalues and eigenvectors to solve this problem, and computing eigenvalues and eigenvectors is complex). The initial $f^{\left(0\right)}$ is initialized randomly. The proposed IMC algorithm is detailed in Algorithm 2.
**Algorithm 2:** IMC algorithm.**Input:**Xcompute $w_{ij}$ by (3),
Randomly initialize $f^{\left(0\right)}$**Repeat**      Compute $f^{\left(n+1\right)}$ via $f_{i}^{\left(n+1\right)}=\frac{\sum_{j}w_{ij}f_{j}^{\left(n\right)}}{\sum_{j}w_{ij}}$**Until**$\left|J^{\left(n\right)}-J^{\left(n-1\right)}\right|$ is less than a prescribed tolerance
or *n* is equal to the maximumnumber of iterations**Output:**f

**Figure 1:**
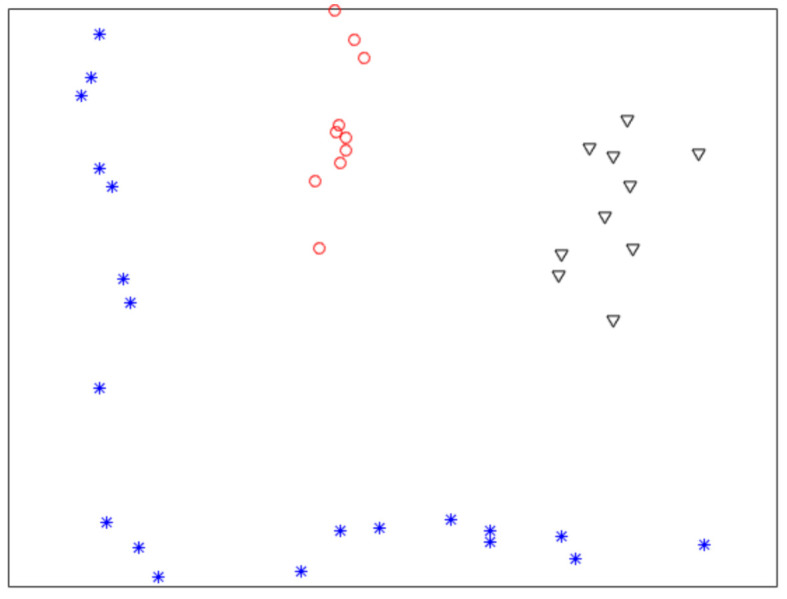
A nonlinearly separable dataset containing three clusters.

**Figure 2:**
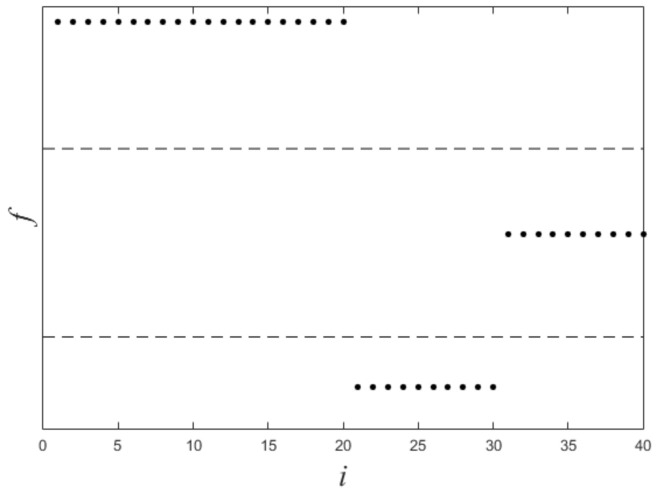
The plot of f for the dataset shown in Figure 1. X-axis means *i* (i.e., the subscript of $x_{i}$), Y-axis means *f*.

**Figure 3:**
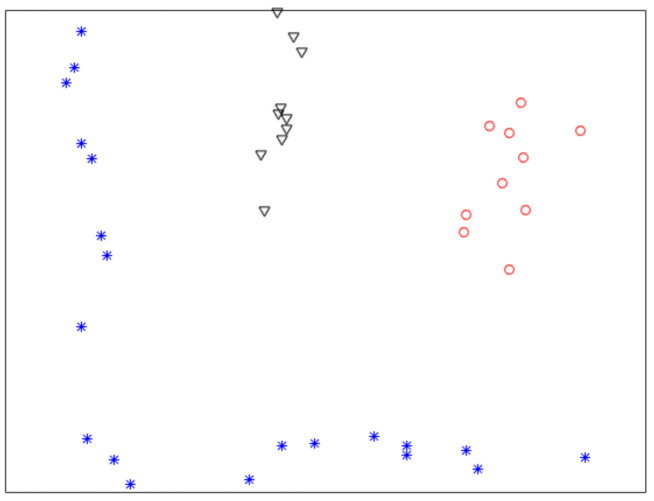
A clustering result of the dataset shown in Figure 1.

Figure 1 shows a nonlinearly separable dataset, and Figure 2 shows its f computed by IMC. From Figure 2 we can see that f is linearly separable, and we can partition it by using thresholding method. Figure 3 shows a final clustering result of IMC, and from it we can see that the clustering result is consistent with the dataset shown in Figure 1.

Next, we consider obtaining the final clustering results by one-dimensional vector f. We partition the one-dimensional vector f into *C* categories by using some basic clustering algorithms (e.g., K-means) or thresholding method:

(8) $$L_{i}=\left\{\begin{array}{cc} 0 & f_{i}<T_{1}\\ \cdots & \cdots \\ c & T_{c}<f_{i}<T_{c+1}\\ \cdots & \cdots \\ C & f_{i}>T_{C} \end{array}\right.$$

where $T_{c}$ is the *c*-th threshold.

## 3. Experiments

**Figure 4:**
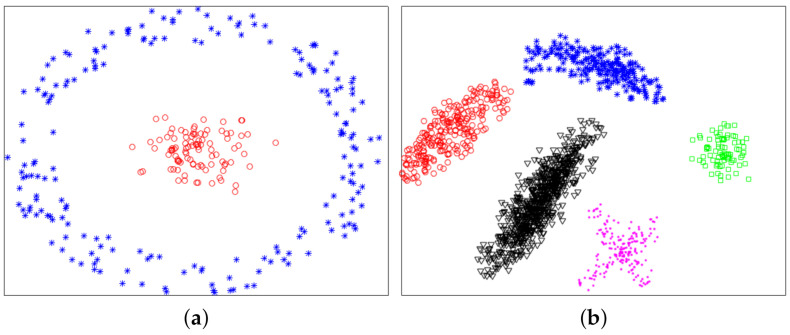
Synthetic datasets. (**a**) Dataset 1. (**b**) Dataset 2.

**Figure 5:**
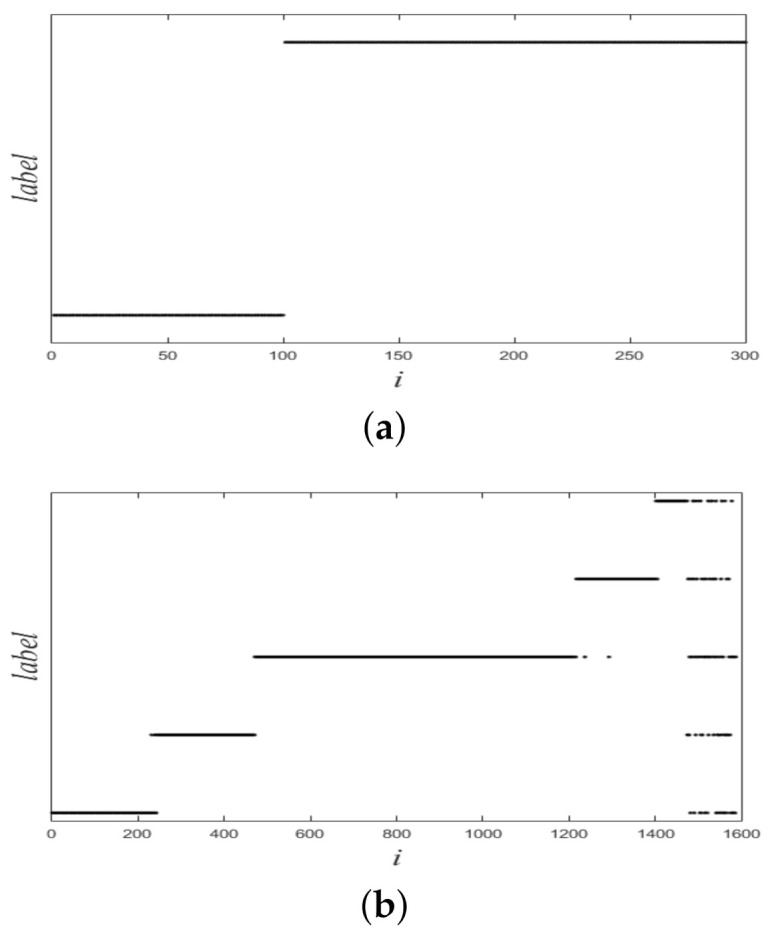
Ground-truth labels of two synthetic datasets. (**a**) Dataset 1. (**b**) Dataset 2.

In this section, we used experiments to evaluate the effectiveness of the proposed method. The variational method indirectly provided a theoretical support for the proposed method. The purpose of experiments was to verify whether the proposed method was valid. We used six datasets: two synthetic datasets (Dataset 1 and 2) and four UCI real datasets. Dataset 2 was from [10]. Dataset 1 and 2 were composed of 300 and 1587 data points from two and five classes, respectively. The two synthetic datasets are shown in Figure 4, and ground-truth labels are presented in Figure 5. UCI real datasets are detailed in Table 1.

All the experiments were implemented using MATLAB 2015a on a standard Window PC with an Intel 2.3 GHz CPU and 8 GB RAM.

### 3.1. Experiments for Synthetic Datasets

In this subsection, we used synthetic datasets to demonstrate the performance of the proposed method for nonlinearly separable datasets. We used KNN graph and set K=10. The σ of (3) was set to 0.1. The maximum number of iterations was 8000 (Note that the computational complexity of (7) was very low, so the algorithm did not take too much time).

**Figure 6:**
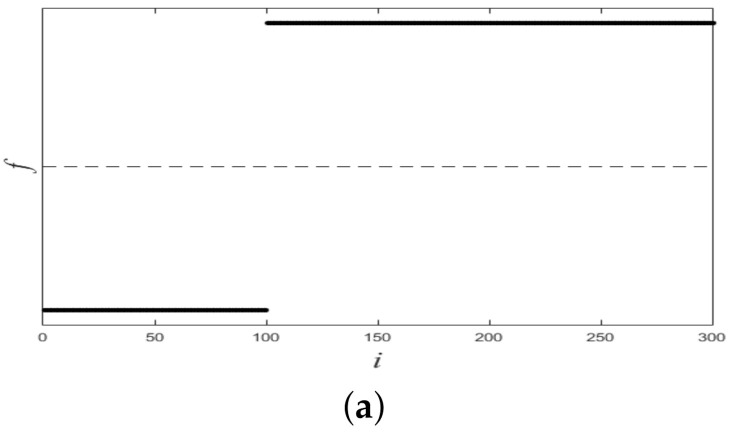

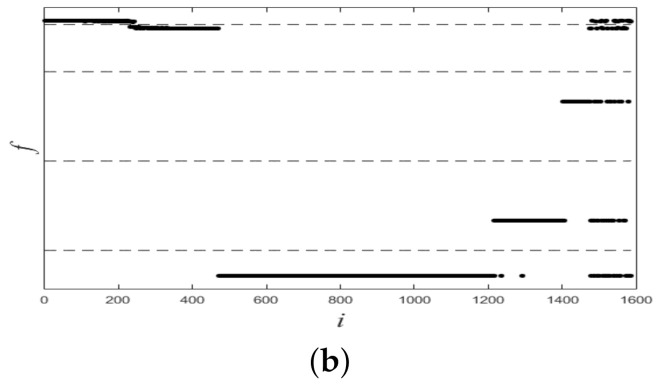
Plots of ***f*** for two synthetic datasets. (**a**) Dataset 1. (**b**) Dataset 2.

**Figure 7:**
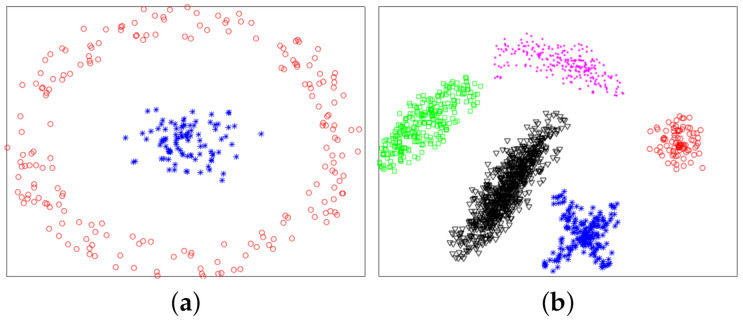
Final clustering results of the proposed IMC. (**a**) Dataset 1. (**b**) Dataset 2.

Figure 6 shows plots of partitioned f on two datasets, and from it we see that all plots of f were linearly separable. Figure 7 shows final clustering results for two datasets, and from it we see that all clustering results were consistent with ground-truth labels, so all clustering results were correct.

### 3.2. Experiment about Convergence

We further carried on to evaluate the convergence of the proposed method. We ran the proposed method 100 times on two datasets with different initial values. If all the results were correct, then the algorithm could be globally convergent. We used NMI [25] as the clustering evaluation metric. NMI is a normalization of the Mutual Information score to evaluate the clustering results between 0 (no mutual information) and 1 (perfect correlation).

Table 2 shows the min, max, and mean of NMI of the proposed method for two datasets. From it we can see that all of clustering results were correct. Thus, the proposed IMC could usually obtain correct clustering results.

### 3.3. Experiments for Real Datasets

In this subsection, we evaluated the performance of the proposed method on real datasets (shown in Table 1). We ran the proposed method (IMC) and spectral clustering (SC) 50 times, and the mean result was retained.

Table 3 shows the mean of NMI and the mean of running time of IMC and SC on two real datasets. The better results in each case are highlighted in bold. From it we can see that:

(1) when the max iteration number was set to 1000 and 2000, IMC needed less running time than SC, but obtained higher NMI than SC;
(2) for different max iteration numbers, IMC obtained different NMI, but all NMI of IMC were higher than those of SC.

## 4. Concluding Remarks

In this article, we propose a novel graph-based clustering algorithm called IMC for solving the clustering problem on nonlinearly separable datasets. We first compute similarities between pairs of data points. Then the proposed IMC maps a nonlinearly separable dataset to a one-dimensional vector by using only one formula. Finally, we use thresholding method or K-means to obtain final clustering results. We use experiments to evaluate the performance of the proposed method on synthetic nonlinearly separable datasets and real datasets, and we also use experiments to demonstrate the convergence of the proposed method. By experiments, on synthetic datasets and little real datasets, the proposed method can provide good clustering results.

We summarize the advantages of the proposed method from the following two aspects.

Theoretical view: (1) the proposed ideal is from variational method. The variational method is well supported by the mathematics theory, so the proposed method is indirectly supported by the theory of variational method; (2) it uses only one formula to solve the problem (spectral clustering requires to compute eigenvalues and eigenvectors to solve this problem, and computing eigenvalues and eigenvectors is complex).

Practical view: the proposed method can obtain good clustering results for synthetic nonlinearly separable datasets and some real datasets.

In the future, we will consider extending IMC by using other graph cut criteria. Moreover, we think one-dimensional data may not represent the structure of large datasets completely, but one-dimensional data is simple (It is both a strength and a weakness). We will consider how to solve this problem.

## Figures and Tables

**Table 1:** UCI real datasets.

Datasets	Codes	Instances	Classes	Dimensions
Iris	D3	150	3	4
Dermatology	D4	358	6	34
Glass	D5	214	6	10
Parkinsons	D6	195	2	23

**Table 2:** The min, max, and mean of NMI of the proposed method on two datasets.

DS	Mean	Min	Max
Dataset 1	1.0000	1.0000	1.0000
Dataset 2	1.0000	1.0000	1.0000

**Table 3:** NMI and running time comparisons of IMC with spectral clustering (SC) on UCI real datasets.

Method	Max	D3		D4		D5		D6	
	Iteration	NMI	Time	NMI	Time	NMI	Time	NMI	Time
SC	−	0.7660	0.0570	0.1116	0.1599	0.331222	0.047160	0.014030	0.014457
IMC	1000	**0.7777**	**0.0111**	**0.1362**	**0.0301**	**0.387883**	**0.014373**	0.014030	**0.011158**
	2000	**0.7716**	**0.0257**	**0.1226**	**0.0653**	**0.359414**	**0.033953**	0.014030	0.030275
	3000	**0.7703**	**0.0432**	**0.1213**	**0.1061**	**0.340673**	0.056648	0.014030	0.049958
	4000	**0.7670**	0.0633	**0.1218**	**0.1426**	0.322012	0.078662	0.014030	0.073234
	5000	**0.7706**	0.0981	**0.1458**	0.1697	0.348434	0.118252	0.014030	0.117675
	6000	**0.7690**	0.1172	**0.1449**	0.2462	0.349533	0.140191	0.014030	0.162995
	7000	**0.7817**	0.1502	**0.1468**	0.3033	0.327493	0.171360	0.014030	0.173535
	8000	**0.7792**	0.2191	**0.1512**	0.3710	0.386487	0.250267	0.014030	0.211110

## Data Availability

Not applicable.

## Abbreviations

The following abbreviations are used in this manuscript:

X	dataset
N	number of data points in a dataset
*H*	dimension of data points
$x_{i}$	i-th data points in a dataset
W	similarity matrix
$w_{ij}$	similarity between $x_{i}$ and $x_{j}$
D	degree matrix
L	Laplace matrix
f	the feature of X
$f_{i}$	i-th value of f
